# Nanoconfined water can orient and cause long-range dipolar interactions with biomolecules

**DOI:** 10.1038/s41598-017-18258-1

**Published:** 2017-12-19

**Authors:** Dirk Hegemann, Nicolas Hocquard, Manfred Heuberger

**Affiliations:** Empa, Swiss Federal Laboratories for Materials Science and Technology, Laboratory for Advanced Fibers, Lerchenfeldstrasse 5, 9014 St.Gallen, Switzerland

## Abstract

Surface properties are generally determined by the top most surface layer also defining how molecules adsorb onto it. By exploring effects due to interactions with deeper subsurface layers, however, long-range interaction forces were found to also significantly contribute to molecular adsorption, in which hydration of the subsurface region is the key factor. Water molecules confined to a subsurface amphiphilic gradient are confirmed to cause these long-range dipolar interactions by preferential orientation, thus significantly changing the way how a protein interacts with the surface. These findings imply future exploitation of an additional factor to modulate adsorption processes.

## Introduction

The interactions of water with solid surfaces are essential for a manifold of biochemical, chemical and physical processes^1^. One particularly important process at the aqueous interface is the adsorption of macromolecules such as proteins which has implications into many application areas such as non-fouling, tissue engineering, biocompatibility or bio-sensing. Control of protein adsorption has so far been limited to the common surface-related interaction forces (surface energy, charging, hydrogen bonding, molecular forces) agreeing that the water structure in the vicinity play important roles^2–5^. In contrast, it was recently discovered that nanoporous films exhibiting a vertical chemical gradient below their surface can significantly change the adsorption of bovine serum albumin^6^. Here we present experimental evidence that this effect of >10 nm long range is generated by nanoconfined, gradient-oriented water. The experimental evidence suggests a phase of water with reduced internal hydrogen-bonding, but with preferential hydrogen bonding and orientation in the specially designed subsurface chemical gradient field of the matrix. The resulting dipolar subsurface phase spawns a dipolar interaction field of long range, which can force adsorbing albumin into a modified conformation. Water confined to a subsurface gradient field thus represents a means of altering molecular interactions with surfaces – far beyond the possibilities of conventional surface chemistry.

Proteins are known to interact with surfaces via different types of interactions and notably change conformation (i.e. denature) in the process of adsorption^7,8^. Not much to a surprise, the hydrophobic-hydrophilic balance of a surface was found to significantly affect the adsorption of proteins^2,9^. For example, on hydrophobic surfaces proteins are commonly found to denature more, thus occupying a larger surface area per molecule than on a more hydrophilic surface.

More recently, there have been noteworthy reports about polar subsurface modifications that significantly affect the amount of adsorbed proteins^10–12^. A notably reduced adsorption of albumin was recently also found to occur above an amphiphilic subsurface gradient, i.e. buried nanometers below the surface^6^. Suitable subsurface gradients have only recently been available. Although amphiphilic gradients are well known to exist naturally inside self-assembled structures (e.g. micelles, membranes, etc.), the here used artificial nano-gradients are made of plasma polymers^6,13^ and are thus fundamentally different because they are stabilized by a network of covalent crosslinks rather than simply amphiphilic equilibrium interactions. We can now present unequivocal evidence that the presence of nanoconfined water inside the plasma polymer subsurface gradient is the key element producing this effect.

## Results and Discussion

A very high stability of the confining plasma polymer (pp) matrix, in the hydrated state, is prerequisite to designing a defined subsurface gradient. Thanks to the high degree of crosslinking achieved in a pp-film, it has become possible to generate embedded amphiphilic nano-gradients that will sustain a much higher energy density in presence of water than amphiphilic equilibrium molecular self-assemblies. The here used pp-films are based on siloxane chemistry, i.e. HDMSO precursor vapor. The matrix was generated by initial deposition of a nominally 50 nm thick hydrophilic base layer (ppSiOx) with the dosed addition of O_2_ gas, followed by a hydrophobic cover layer (ppHMDSO) of varying thickness, *D*, of several nanometers^6^.

### Hydration of gradient structure

**Figure 1:**
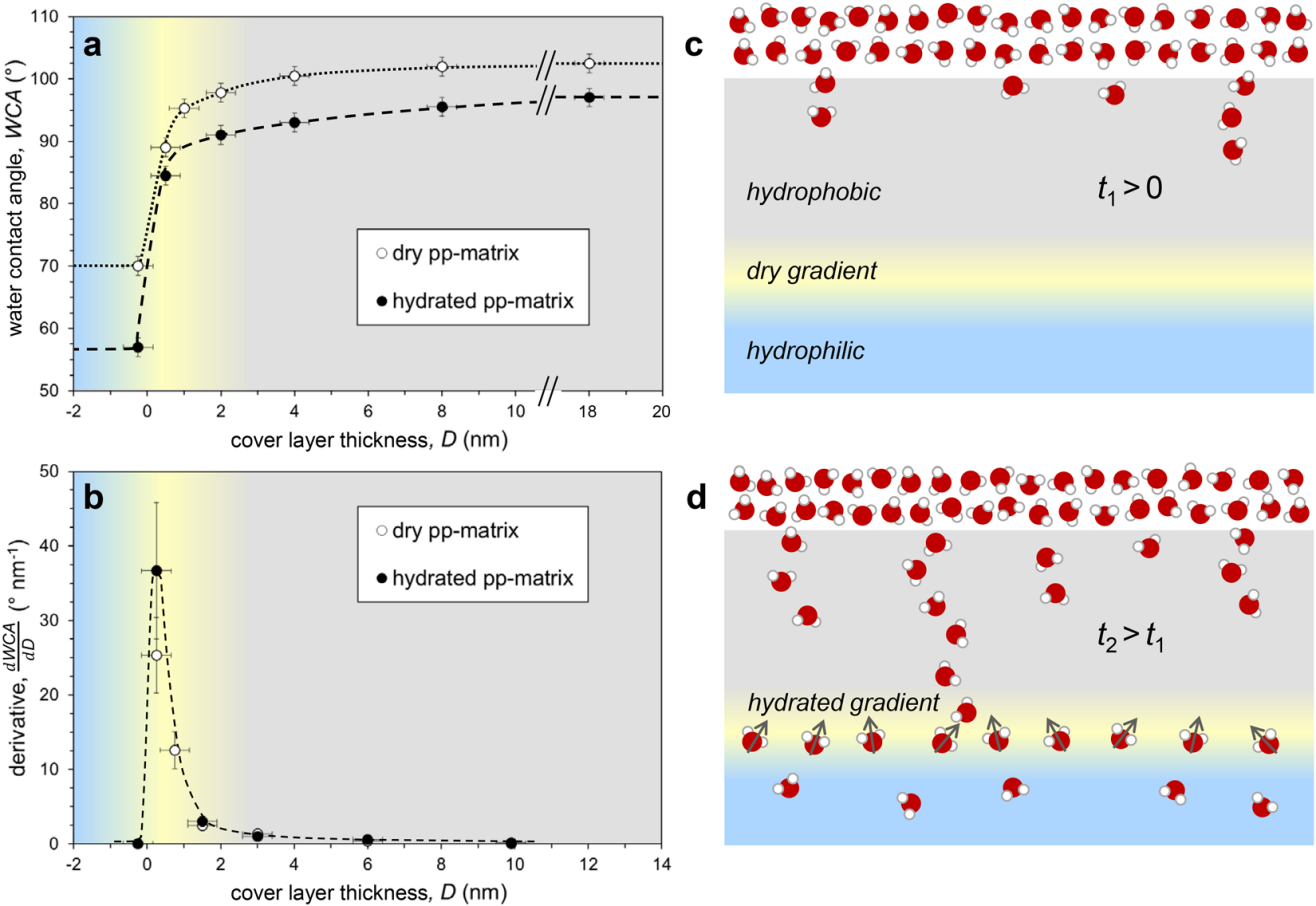
Hydration of subsurface hydrophobic-to-hydrophilic gradients. (**a**) Water contact angles (*WCA*) measured on the hydrophilic base layer (blue) with hydrophobic termination (gray) of different cover layer thickness, *D*, (0–18 nm). Hydrated plasma polymer films, i.e. immersed in water for 16 hrs, revealed lower contact angles. (**b**) Derivative $\frac{{dWCA}}{{dD}}$ emphasizing the location and width of the nano-gradient (yellow); this gradient is remarkably independent of the hydration state; the gradient represents the location of the chemical gradient field that orients confined water. (**c**) Water diffusion into a hydrophobic matrix is expected to produce a ramified front (at time *t*_1_). (**d**) Water (with a dipole moment) that hydrates the amphiphilic transition region becomes oriented due to the asymmetry of available hydrogen bonds (quasi equilibrium at time *t*_2_).

The hydration of a stratified pp-matrix is known to be substantially facilitated via incipient silanol (Si-OH) groups in the network^14^. The interaction between water and the pp-matrix can be readily characterized via surface water contact angle (WCA) measurements obtained at different stages of the matrix formation process. Using this depth-resolved WCA (drWCA) approach, we can elucidate the hydrophilic-to-hydrophobic transition (Fig. 1a), which is otherwise buried nanometers below the surface.

The drWCA method can also be used to assess different stages of hydration of the matrix; for example, to study the state of equilibrium hydration, where the sample is stored 16 hours in water and dry blown seconds before the WCA measurement. One can detect significantly lower contact angles on such equilibrium-hydrated surfaces than on dry-stored surfaces (two curves in Fig. 1a). This illustrates that the drWCA method can deliver valuable information about the subsurface hydration capacity inside the plasma polymer films^13^. As expected, the hydrophobic variant of the pp-film is hydrated to a lesser degree, resulting in a smaller WCA difference between dry- and hydrated states. We could independently confirm and quantify these differences in pp-subsurface hydration behavior using neutron reflectivity (NR) measurements^14^.

Figure 1b displays the derivative of the drWCA, which is a measure for the strength of the chemical gradient field that the confined water is exposed to. The related (Young’s equation) change of surface energy per depth in the gradient has the physical unit of an energy density [J m^−3^]. The region of high energy density is thus asymmetrically distributed over a narrow region of 1–2 nm in depth. This gradient region is formed and stabilized during the plasma polymerization via interaction of high energy ions with the base-layer surface during initial growth of the more hydrophobic cover layer.

Water intrusion into a hydrophilic porous material is expected to proceed with a flat front, whereas penetration into a hydrophobic material (>90°) may produce a ramified front with a fingered morphology^15^. While the exact channel geometry has little influence^16^, hydration always continues through the least resistance path (nanochannel)^17^, as illustrated in Fig. 1c. Equilibration for 16 hours in water was experimentally found to ensure hydration across all hydrophobic top coatings used in this study. It is reported that water molecules show a net orientation of their O–H groups pointing towards the hydrophobic layer, which is affected by hydrogen bonding, ionic strength, and pH value^5,18^. Once the water intrusion has reached the gradient, the more hydrophilic matrix of the base layer ensures a faster lateral spread as sketched in Fig. 1d. Such nanoconfined water might reorient depending on the CH_3_ and OH group density within the hydrophobic-to-hydrophilic gradient region affecting the hydrogen-bond structural dynamics of water. Previous NR measurements quantified the equilibrium water volume fraction in the subsurface of a reference hydrophilic matrix between 10–25%^14^. Hence, the effective water density is at least 4–10 times lower in the gradient matrix than in bulk water, yielding an average distance 4.5–6 Å between water molecules. At such intermolecular distances, the orientation constraints imposed by water-water hydrogen bonding are largely replaced by the hydrogen bonds formed with the confining matrix. In a stable, hydrophobic-to-hydrophilic chemical gradient, the distribution of hydrogen bonding sites is expected to induce a preferred orientation of the nanoconfined water as indicated in Fig. 1d.

### Protein adsorption on gradient structure

**Figure 2:**
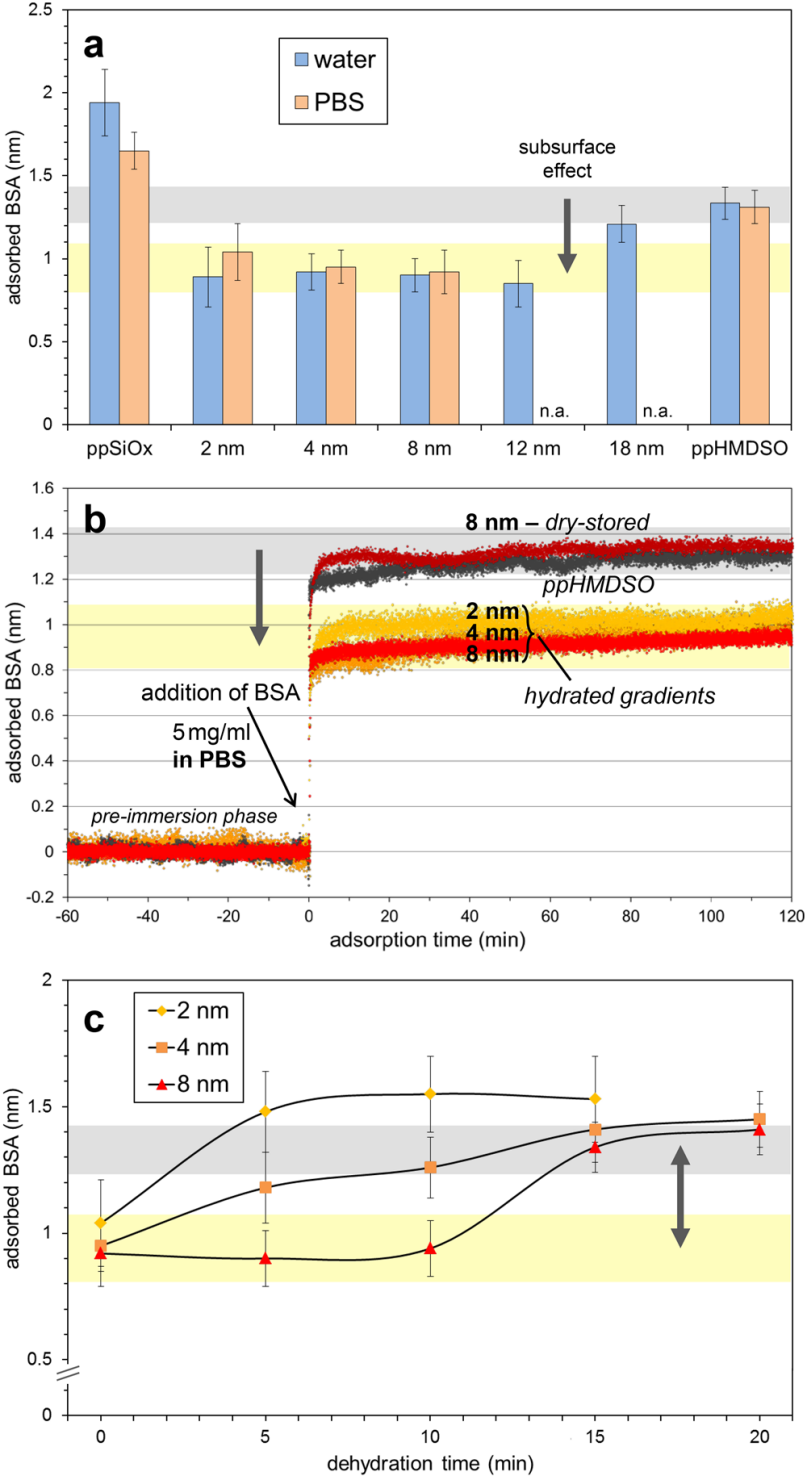
Optical thickness of adsorbed BSA probing the protein-surface interaction as a function of salt concentration, subsurface hydration state and cover layer thickness (range). (**a**) Adsorbed BSA on hydrated gradients with different cover layer thickness, *D*, (2–18 nm) immersed in 5 mg mL^−1^ BSA solution in water (pH 6.8, Debye length ~300 nm) or PBS (ionic strength 0.15 M; pH 7.4, Debye length ~0.86 nm). A reduced protein adsorption (yellow band) is observed below the reference value (gray band) obtained on the hydrophobic plain matrix. The adsorbed thickness on the gradient-free matrices, namely the hydrophilic base layer (far left: ppSiOx) or the hydrophobic top layer (far right: ppHMDSO), are shown for reference. (**b**) Time-resolved TInAS protein adsorption curves showing that dry-stored gradients exhibit no effect, but equilibrium hydrated subsurface gradients reduce protein adsorption. (**c**) Dehydration series of previously equilibrium hydrated matrices showing a loss of the effect with dehydration time (gray and yellow band as in (**a**)). The loss of the effect is delayed for thicker cover layer thickness, indicating that it is specifically dependent on the remaining hydration at the subsurface gradient.

To this end we use hydration/dehydration schemes of the pp-matrix and variation of the cover layer thickness, *D*, in a range from 2–18 nm (Fig. 2a) to prove the key role of gradient-confined water as well as the long-range nature of the observed effect. The amount of adsorbed protein from a 5 mg mL^−1^ solution of bovine serum albumin (BSA) serves again as probe for the modified surface interactions. Although, unspecific about the type of interaction, this method is sensitive to detect changes in the sum of all interactions with the surface. We use the Interferometric Adsorption Sensor (TInAS)^6,19^ to measure the thickness of the adsorbed protein film at sub-Ångstrom resolution, in which both distilled water and phosphate buffered saline (PBS) was considered as media to discriminate electrostatic from dipolar effects.

The gray band in Fig. 2a indicates the reference adsorbed amount of protein on the plain hydrophobic pp-matrix without subsurface gradient. The yellow band indicates the reduced amount of adsorbed BSA in presence of a hydrated subsurface gradient – the subsurface effect. We can readily see in Fig. 2a that the effect is detected up to a cover layer thickness of >10 nm, which illustrates its remarkable long-range nature. Indeed, only electrostatic or dipolar interactions are known to be of similar range^20^. Furthermore, the independence of this effect on a significant change of Debye length (screening depth) by replacing water (~300 nm) with PBS (~0.86 nm) rules out a simple electrostatic interaction, while the water structure at the surface is affected^21,22^. At this point we note a small difference in amount of adsorbed protein on the plain hydrophilic ppSiOx matrix as a function of salt concentration; this is expected due to the acquisition of a negative charge hindering the adsorption of negatively charged BSA molecules^9,23^.

Figure 2b displays the TInAS data measured during adsorption of BSA on the hydrophobic cover layer matrix with/without subsurface gradient. Remarkably, the dry-stored cover layer with subsurface gradient adsorbed similar amounts of BSA as the same matrix without such gradient, meaning that the effect is not observed with a dry subsurface gradient. The effect of reduced protein adsorption is thus only activated in ample presence of water inside the subsurface gradient, in which hydration is largely independent of salt concentration^24^. Figure 2c illustrates that the activation of the effect is perfectly reversible by dehydration of the gradient via rapid expulsion of water from the narrow confinement^24^. The dehydration rate and concurrent loss of the effect scale with the hydrophobic cover layer thickness, *D*, agreeing well with findings that the evaporation rate through nanochannels varies proportionally with 1/*D* ^25^. Hence, hydration of the buried gradient – not the cover layer – is the key for activation. We recall that all samples shown in Fig. 2b have the same surface chemistry of the hydrophobic ppHMDSO termination and conclude that hydration of the buried gradient reversibly generate a long-range interaction by orientation of water molecules in the nanoconfined region.

To measure the changes of surface potential, we have measured the pp-matrix via Kelvin probe AFM both on dry-stored and equilibrium hydrated pp-gradient structures. The here measured contact potential difference between conductive tip and sample depends on the work function, the electronic structure and dipoles at the surface^26,27^. A remarkable shift in contact potential of ~500 mV was observed between a dry-stored and an equilibrium hydrated subsurface of the same matrix with a gradient 8 nm below the surface (measured directly after dry blowing). Since all other matrix parameters were kept the same, this finding is indicative of a significant change in surface charging or the dipole field at the surface. Analogue to the dehydration experiment shown in Fig. 2c the Kelvin potential difference was found to gradually diminish with dehydration time, i.e. to about 300 mV after 20 min. This result again demonstrates that the measured effect specifically and reversibly depends on the hydration state of the subsurface gradient.

### Strength and range of dipolar surface interactions

How can nanoconfined water in a subsurface gradient generate such dipole field? Although water molecules have a significant dipole moment of *µ*_*H2O*_ = 1.85 D, bulk water cannot readily be oriented. The reason is that hydrogen bonds between neighboring water molecules impose tetrahedral dipole orientations with overall vanishing dipole moment. Still, it has recently been reported that water, when confined in beryl crystal, exhibits ferroelectric properties^28^ because an intermolecular distance of 5 Å is sufficient to alleviate the tetrahedral orientation from bulk water hydrogen bonding. A similar situation arises when water is confined inside a carbon nanotube^29,30^. We note that the average spacing between water molecules in our pp-matrix is of similar magnitude, which could allow a ferroelectric phase of water to exist. The nano-gradient imposes a field on the confined water that could collectively orient the molecular dipoles normal to the plane of the gradient. This explanation predicts a macroscopic dipole field emerging from the buried gradient. Such orientation would entail a high energy density and an effective repulsion between water molecules. Along these lines, this dipolar field could also affect the orientation of the ferroelectric water in the remaining pp-matrix and even alter the water structure above the surface.

There is experimental evidence that albumin has an extraordinary permanent dipole moment of *µ*_*BSA*_ = 384 D in solution and a characteristic dipolar re-orientation time in the order of 1 µs^31^. It is thus not surprising that albumin interacts with a surface dipolar field. It can be expected that the orientation and likely also the conformation of the adsorbing protein is affected, which influences the thickness of the adsorbed film in the way observed here.

Proteins can interact with surfaces via different interactions, including Van der Waals, electrostatic, dipolar, amphiphilic forces, water structure or specific interactions (i.e. mixture of above). Using protein adsorption as a probe for changes in the interaction is thus, a priori, an undifferentiated tool to identify changes in one specific kind of the above listed interactions. Nevertheless, we can rely on the fact that different interactions have a distinct range of action. Notably, only electrostatic or dipolar interactions have a range that goes beyond >1–2 nm. The electrostatic interaction can be ruled out here as cause, since the effect of reduced protein adsorption is completely unaffected by a significant change of salt concentration, i.e. from deionized water to 1x PBS solution. Therefore, the data clearly suggest that the effect is caused by a dipolar interaction, involving oriented molecular dipoles.

Let us compare the experimentally observed range $\gtrsim$10 nm of the effect with the theory of intermolecular forces^20^. We may generally describe the interaction of a probing molecule (e.g. protein) with a surface as the sum (integral) of pair-interactions by invoking a number density of molecules. This is a commonly applied approximation, which is based on the assumption of additivity. The underlying pair potentials can be described by a Mie potential with a negative (attractive) and a positive (repulsive) term.

1 $$w(r)=-\frac{{C}_{1}}{{r}^{n}}+\frac{{C}_{2}}{{r}^{m}}$$

A prominent example for eq. (1) is the parameter set *n* = 6, *m* = 12, *C*_1_ = 10^−77^ Jm^6^ and *C*_2_ = 10^−1^ ^34^ J m^12,^ which describes the Lennard-Jones potential.

The exponent, *n*, of the negative term determines the effective range of the attractive interaction; it depends on the nature of the interaction; the smaller *n*, the larger the interaction range (Table 1).

**Table 1:** Listing typical exponents of the Mie potential used to describe different types of molecular interactions.

Exponent *n*	Type of interaction
1, 2	charge-charge, charge-(fixed) dipole, hydrogen bond
3	(fixed) dipole-(fixed) dipole
4	charge-(free) dipole, charge-neutral
6 (Van der Waals)	neutral-neutral (London dispersion), (free) dipole-(free) dipole (Keesom energy), (free) dipole-neutral (Debye energy)

Abbreviations: neutral = polarizable molecule without net electrical charge; charge = molecule with net electrical charge; (free) dipole = freely rotating molecule with dipole moment; (fixed) dipole = molecule with dipole moment and fixed orientation.

The interactions with *n* = 6 belong to the Van der Waals type and are short-range, i.e. typically $\lesssim$3 nm. The only non-electrostatic type of long-range interaction that can explain the experimentally observed effect on adsorbing BSA is involving, in one way or another, fixed dipole moments; effects due to charging could be excluded (as discussed above).

**Figure 3:**
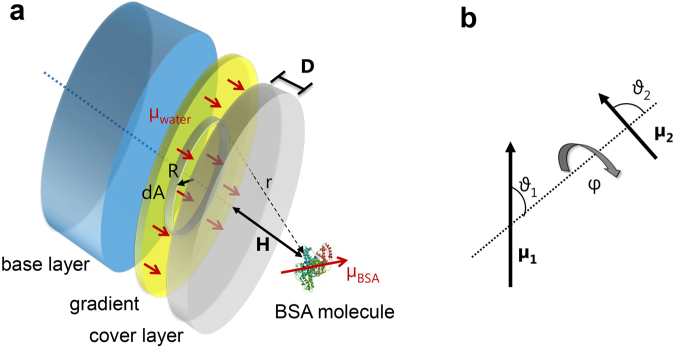
Model of interaction forces. (**a**) Schematic of three layer model used to estimate the interaction potential of a (rigid) BSA molecule at a height, H, above the surface. Below the surface is the subsurface gradient with a molecular layer of dipole-oriented water. The cover layer thickness, *D*, determines the depth of the oriented layer. It is modeled as a (5% of areal point density) 2D sub-monolayer of oriented water molecules. We assume additivity and integrate the dipole-dipole pair potential using infinitesimal rings, *dA*, of radius, *R*, that are coaxial with the surface normal of the BSA molecule. (**b**) The used angles and dipole moments are defined as shown.

Let us now estimate the interaction potential of bovine serum albumin (BSA) at a height, *H* + *D*, above a sub monolayer of oriented water molecules (gradient) as illustrated in Fig. 3. We want to calculate the interaction energy by modeling dipole oriented water molecules arranged in a single layer at the gradient location.

Water molecules have a dipole moment of *µ*_*water*_ = 1.85 D and albumin exhibits an even more important overall dipole moment of *µ*_*BSA*_ = 384 D, which causes the experimentally observed dielectric dispersion of the rotating molecule around 1 MHz (1 Debye [D] ~ [3.33564 10^−30^ Cm])^31^. For this first estimation, we will assume that the BSA molecule does not deform.

The pair potential for the dipole-dipole interaction is given as:

2 $$w(r)=-\frac{{\mu }_{water}{\mu }_{BSA}}{4\pi {\varepsilon }_{0}{r}^{3}}f({\vartheta }_{1},{\vartheta }_{2},\phi )$$

with an angular dependent function as:

3 $$f({\vartheta }_{1},{\vartheta }_{2},\phi )=2\,\cos \,{\vartheta }_{1}\,\cos \,{\vartheta }_{2}-\,\sin \,{\vartheta }_{1}\,\sin \,\vartheta {\phi }_{2}\,\cos \,\phi $$

We integrate over coaxial, rotational symmetric surface areas, *dA*:

4 $$dA=2\pi RdR,$$

*R* is the radius of the infinitesimal ring, with oriented water molecules in the infinitesimal ring area, *dA*, at a distance, *r*, from the BSA:

5 $$r=\sqrt{{(H+D)}^{2}+{R}^{2}},$$

*H* is the height or normal surface distance of the BSA molecule. We assume an areal density of oriented water, *σ* = 5% ML in the gradient; with 100% monolayer density [ML]_bulk water_ ~ [1.23 10^19^ m^−2^], as approximated from a 2D cubic lattice with an O–O spacing of 2.85 Å.

The total interaction potential can then be written as an integral:

6 $$W(H)=-\frac{{\mu }_{water}{\mu }_{BSA}\sigma }{4\pi {\varepsilon }_{0}}{\int }_{R=0}^{R=a}dR\frac{2\pi R}{{\sqrt{{(H+D)}^{2}+{R}^{2}}}^{3}}f({\vartheta }_{1},{\vartheta }_{2},\phi )$$

**Figure 4:**
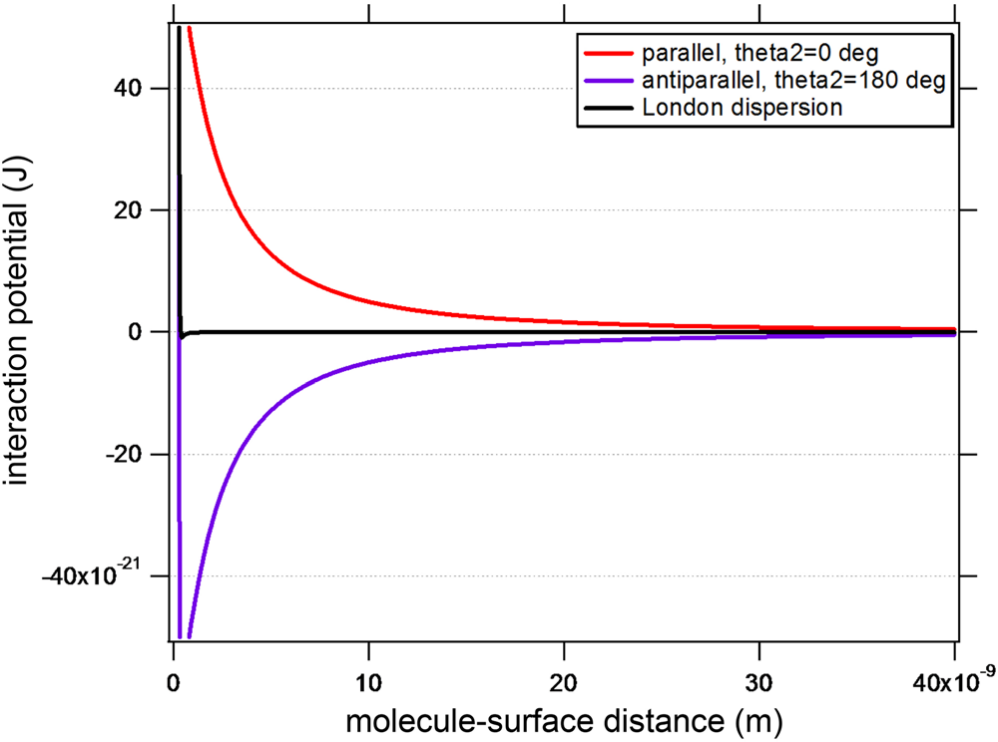
Interaction potentials, *W*(*H*), of BSA with a 5% subsurface monolayer of oriented water. The cover layer thickness was *D* = 4 nm in this calculation. Different curves show the influence of the BSA orientation, *ϑ*_2_.

We have used numerical integration (of sufficient size i.e. *a* = 10^−2^ m) to calculate the resulting interaction potentials according to eq. (6), as a function of molecule-surface distance, *H*, cover layer thickness, *D*, as well as BSA dipole orientation, *ϑ*_2_. Figure 4 displays the calculated interaction potentials, *W*(*H*), with added Pauli exchange repulsion (*m* = 12) at contact. As expected, the dipole-dipole interaction dominates over the London dispersion interaction (black color) and has a range of ≳10 nm, like the effective distances observed in the experiment. Generally, the negative slope of the interaction potential *F*(*H*) = −*dW*(*H*)/*dH* corresponds to the force experienced by a BSA molecule near the surface.

In summary, we note the following for the fixed dipole-dipole interaction:

(i) it is of long range ≳10 nm and stronger than the dispersion interaction.
(ii) it is attractive for anti-parallel orientation of water and BSA, *ϑ*_2_ = 180°.

**Figure 5:**
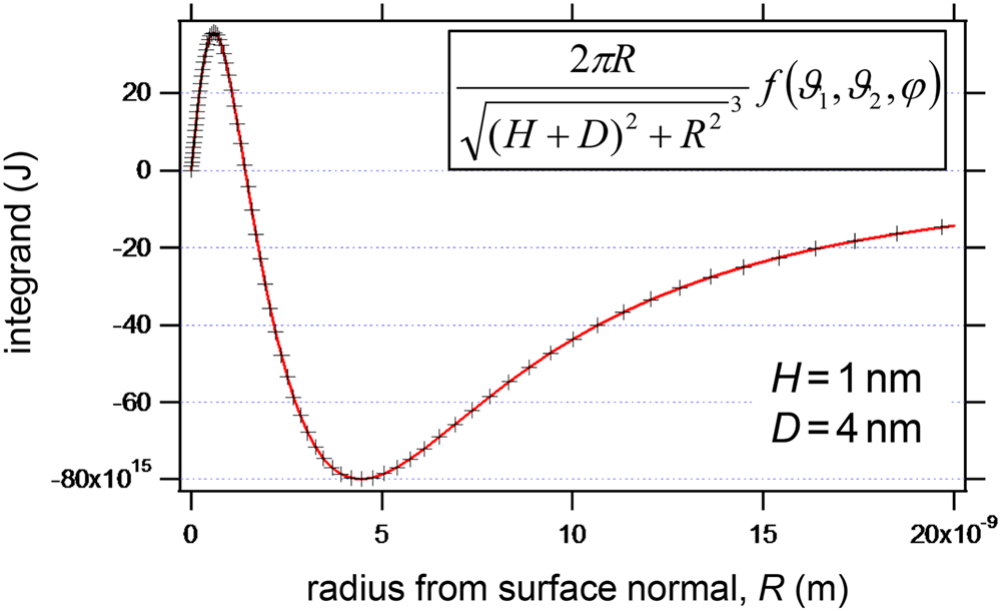
Interaction potential integral. The integrand is plotted versus the integration variable *R*, which is the radius of the infinitesimal ring.

To understand why the antiparallel dipole orientation is the attractive one, let us consider the integrand at a distance *H* = 1 nm plotted as a function of the integration variable, *R*. We note from Fig. 5 that, while the sum of the potential is attractive (negative) at larger distance from the normal, there is a rather strong repulsion from the areas that are closer to the normal of the BSA molecule due to the anti-parallel dipoles. In reality, the BSA molecule is not point-like but roughly 14 × 4 × 4 nm^3^ in dimension, and it is also not perfectly rigid^32^. The macromolecule will deform and dipolar sub-domains will realign individually due to the strong gradients of the interaction potential in the vicinity of the surface. The resulting enhanced change in conformation of the flexible BSA molecule in the ≳10 nm dipolar surface vicinity may hold the key to understanding the significantly reduced adsorbed mass seen by TInAS.

We also note that the here presented interaction potential calculations are oversimplified and based on the assumption of pair potential additivity, which is strictly not correct. Furthermore, no medium (water molecules) above the surface were considered (i.e. vacuum). More elaborate calculations using MD/MC simulations are thus required to correctly simulate surface-vicinity force fields in this system. Open questions include the potentially amplifying effect of ferroelectric water in the base- and cover layers or the modifications of the water structure in the vicinity of the surface, as well as the rotational and conformational response of BSA in this highly graduated dipolar interaction potential.

In conclusion, we think that the control over ferroelectric or dipolar properties by design of stable pp-matrices and hydrated subsurface gradients offers new strategies of tailoring and studying surface-based reactions in chemistry and biology. The independence of this approach from the surface chemistry will allow optimization of multiple properties. In particular, we anticipate that subsurface dipolar fields, due to their profound influence on protein conformation, will have significant influence on the binding activity or enzymatic activity of surface-adsorbed proteins. Hence, an additional factor to control adsorption processes is at hand suited to regulate protein and subsequent cell adhesion.

## Methods

### Plasma Polymer Deposition

A capacitively-coupled, asymmetric plasma reactor was used for the deposition of plasma polymer films (PPFs). The radio-frequency-driven (13.56 MHz) electrode (21 × 70 cm^2^) was mounted inside the reactor chamber with 8 cm distance to the chamber wall. A gas showerhead facing the electrode was used to provide a uniform gas inlet, while a pumping system of a rotary and a roots pump sustained a base pressure of 0.2 Pa^13,14^. The liquid compound hexamethyldisiloxane, (CH_3_)_3_-Si-O-Si-(CH_3_)_3_ (purchased from Fluka) was vaporized and introduced with a fixed gas flow rate of 4 sccm (*standard cubic centimeter per minute*) mixed with 20 sccm of argon to deposit hydrophobic ppHMDSO PPFs using a power input of 50 W. Additional admixture of 40 sccm oxygen at a power input of 100 W was applied to largely remove hydrocarbons (forming CO_x_ and H_2_O) during the plasma deposition process resulting in hydrophilic ppSiOx PPFs. Pressure was kept constant at 7 Pa for all experiments.

For the gradient deposition, at first, 50 nm of ppSiOx was coated on the substrate (Si wafers, glass slides and TInAS sensors) followed by the deposition of the hydrophobic ppHMDSO termination with varying thickness (*D*, cover layer thickness, 0.5–18 nm). The plasma was shut off in between the two coating sequences to change the gas composition and power setting (~10 s) while maintaining the working pressure. A narrow interphase of ~1–3 nm is thus formed by plasma interaction during the deposition of the cover layer. Chemical composition, stability and hydration of such PPFs were previously established^6,13,14^.

### Albumin Solutions (in water and PBS)

For each adsorption measurement a freshly prepared solution of 5 mg mL^−1^ powdered bovine serum albumin (BSA; purchased from Sigma-Aldrich, and stored in a fridge at 4 °C) in bidistilled water (pH 6.8) or PBS (ionic strength 0.15 M; pH 7.4) was prepared and equilibrated at a temperature of 20 °C (for 1 hr) prior to its use.

### (Depth-Profiling) Water Contact Angle Measurement

WCA (Krüss, DSA25) was measured using the sessile drop method by depositing drops (2 μL) of water (CHROMASOLV, for HPLC, Sigma-Aldrich). For depth-resolved measurements (drWCA) plasma coatings (ppSiOx) with varying thickness (0–50 nm) of the hydrophobic termination (ppHMDSO) were prepared on glass slides and WCA were recorded at five different positions on the sample surface.

### Protein adsorption measurement

The adsorption of albumin on plasma polymer films was measured using the Transmission Interferometric Adsorption Sensor (TInAS)^19^, which represents a label-free affinity detection based on white light interferometry. The adsorption of a molecule to the water-exposed surface of the sensor causes a shift in the phase of light, which is detected as a wavelength shift of the constructively interfering fringes. The sensing surface is mounted inside a small fluid cell with the possibility to pass different solutions over the surface at constant temperature 20.00 ± 0.05 °C. Prior to the introduction of protein solution at *t* = 0, the surfaces were equilibrated in ultra-pure water or standard phosphate-buffered saline (1x PBS). Assuming a refractive index of *n* = 1.55 for the adsorbed film of proteins, one can determine the nominal thickness, Δ, of the adsorbed layer in nanometers from the measured shift of the interference fringes. This value could also be used to calculate the adsorbed mass per surface area via the refractive index difference of the adsorbed protein, *n*_*BSA*_, and the solvent, *n*_*H2O*_, divided by the derivative of solution refractive index versus BSA concentration, *dn*/*dc* ^33,34^:

$$M={n}_{BSA}-{n}_{H2O}/(dn/dc)\cdot {\rm{\Delta }};\,\,{\rm{for}}\,{\rm{BSA}},dn/dc\approx 0.182\,c{m}^{3}\,{g}^{-1}.$$

### Kelvin probe measurements

The Kelvin probe AFM operates in a non-contact dynamic mode, where the conducting tip is subject to vertical oscillations and thus sensing the tip-surface capacitance. In addition to the AC excitation voltage, a DC offset voltage is applied to the cantilever to balance the local contact potential difference between tip and surface. The result is a lateral image of the surface potential or work function at millivolts resolution. The surface potential is influenced by local charges or dipole moments in the vicinity of the probe. Since the Kelvin probe is a non-contact method, a thin adsorbed water film cannot account for the large effects (≫50 mV) measured during dehydration^35^. Typical surface potential differences measured by Kelvin probe AFM on various polymer mixed surfaces are in a range 0–1000 mV^36^.
